# M-CSF supports medullary erythropoiesis and erythroid iron demand following burn injury through its activity on homeostatic iron recycling

**DOI:** 10.1038/s41598-022-05360-2

**Published:** 2022-01-24

**Authors:** John G. Noel, Seth W. Ramser, Lori Pitstick, John P. Bonamer, Bryan Mackenzie, Katie G. Seu, Theodosia A. Kalfa, Jose A. Cancelas, Jason C. Gardner

**Affiliations:** 1 Division of Pulmonary, Critical Care and Sleep Medicine, University of Cincinnati College of Medicine, Cincinnati, 45267 USA; 2 Department of Pharmacology and Systems Physiology, University of Cincinnati College of Medicine, Cincinnati, 45267 USA; 3 Cancer and Blood Diseases Institute, Cincinnati Children’s Hospital Medical Center, Cincinnati, 45229 USA; 4 Divisions of Pathology and Experimental Hematology and Cancer Biology, Cincinnati Children’s Hospital Medical Center, Cincinnati, 45229 USA

**Keywords:** Erythropoiesis, Cytokines, Haematopoietic cell growth factors, Inflammation, Trauma

## Abstract

M-CSF receptor signaling supports the development and survival of mononuclear phagocytes and is thought to play a role in post burn anemia by promoting myeloid lineage bias. We found M-CSF secretion was increased in burn patients and a murine model of post burn ACI, so we neutralized M-CSF in ACI mice to determine if erythropoiesis was improved. Instead, M-CSF blockade further impaired erythropoiesis and erythroid cells access to iron. M-CSF blockade enhanced inflammatory cytokine secretion, further increased systemic neutrophil counts, and led to tissue iron sequestration that was dependent, in part, on augmented IL-6 secretion which induced hepcidin. Deleterious effects of post burn M-CSF blockade were associated with arrest of an iron recycling gene expression signature in the liver and spleen that included Spi-C transcription factor and heme oxygenase-1, which promote heme metabolism and confer a non-inflammatory tone in macrophages. Hepatic induction of these factors in ACI mice was consistent with a recovery of ferroportin gene expression and reflected an M-CSF dependent expansion and differentiation of Spi-C+ monocytes into Kupffer cells. Together, this data indicates M-CSF secretion supports a homeostatic iron recycling program that plays a key role in the maintenance of erythroid cells access to iron following burn injury.

## Introduction

The anemia of critical illness (ACI) is a rapidly developing and persistent anemia associated with inflammation that occurs in nearly every patient in an intensive care unit for more than 1 week^1,2^. ACI accounts for the majority of transfusions a burn patient receives^3^ and even with the adoption of conservative hemoglobin targets, patients with 20–60% total body surface area burns typically receive an amount of blood that is roughly equivalent to vascular volume^4^. There are no alternatives to transfusion because erythropoietin (EPO)^5–8^ and iron^9^ supplements do not promote effective erythropoiesis in trauma victims. Resistance to EPO and iron supplements is a hallmark of inflammation driven anemia, thus a better understanding of the inflammatory mechanisms in post burn ACI could lead to clinical interventions that reduce transfusions.

Utilizing a murine burn model of ACI, we previously identified G-CSF secretion as a mechanism that leads to impaired red blood cell production in the bone marrow^10,11^. Post burn G-CSF blockade largely rescued erythroid cellularity in the marrow and markedly attenuated an impairment of EPO signaling^10^. However, we suspected involvement of other mediators because G-CSF blockade did not restore peripheral red blood cells counts in ACI mice. Previous reports have suggested that an ineffective response to EPO following burn injury may be due to myeloid biased lineage commitment which diminishes the pool of cells available to respond to EPO in the marrow and in turn reduces the number of mature red blood cells in circulation^12–15^. Increased expression of M-CSF receptors in hematopoietic stem cells and progenitors driven by adrenergic receptor signaling is a suspected mechanism of burn induced myeloid bias^14,15^, but no prior study has explicitly tested the role of M-CSF receptor or M-CSF as a mediator of post burn ACI.

M-CSF/M-CSF receptor signaling also plays a critical role in an adaptive response to heme burden following erythrocyte destruction that involves induction of Spi-C transcription factor (Spic) and heme oxygenase 1 (Hmox1), which promote differentiation of monocytes into iron recycling macrophages and heme metabolism respectively^16,17^, and which are known to promote a non-inflammatory phenotype in macrophages^18–20^. Iron recycling macrophages rely on Hmox1 for metabolism of heme; when Hmox1 is absent or saturated, intracellular heme accumulates and induces apoptosis in macrophages^21,22^. The critical role of Spi-C and M-CSF in the development of iron recycling macrophages is exemplified by the absence of red pulp macrophages in Spi-C deficient mice^23^ and severe reduction of these cells in M-CSF deficient (Op/Op) mice^24^. The role of heme metabolizing macrophages following burn injury has not been extensively studied but is likely important as hypoferremia^25,26^ and enhanced erythrocyte destruction is characteristic of burn injury^27–30^. Further, at least one prior study recognized Kupffer cells were depleted within hours of burn injury in association with erythrophagocytosis and suggested infiltrating monocytes may compensate by transforming into Kupffer cells^31^, reminiscent of the M-CSF dependent adaptation to increased heme burden that is now understood to occur in conditions that damage red blood cells^16^.

In this study, we quantified increased M-CSF secretion in burn patients and a murine model of post burn ACI. Since M-CSF could promote myeloid bias that leads to ACI or homeostatic adaptation that supports recovery from ACI, we tested the role of M-CSF using a neutralization strategy. Our results indicate M-CSF/M-CSF receptor signaling supports homeostatic iron recycling and a non-inflammatory tone in macrophages that plays a key role in the maintenance of erythroid cells access to iron following burn injury.

## Results

### Burn injury induces M-CSF secretion in humans and mice

**Figure 1:**
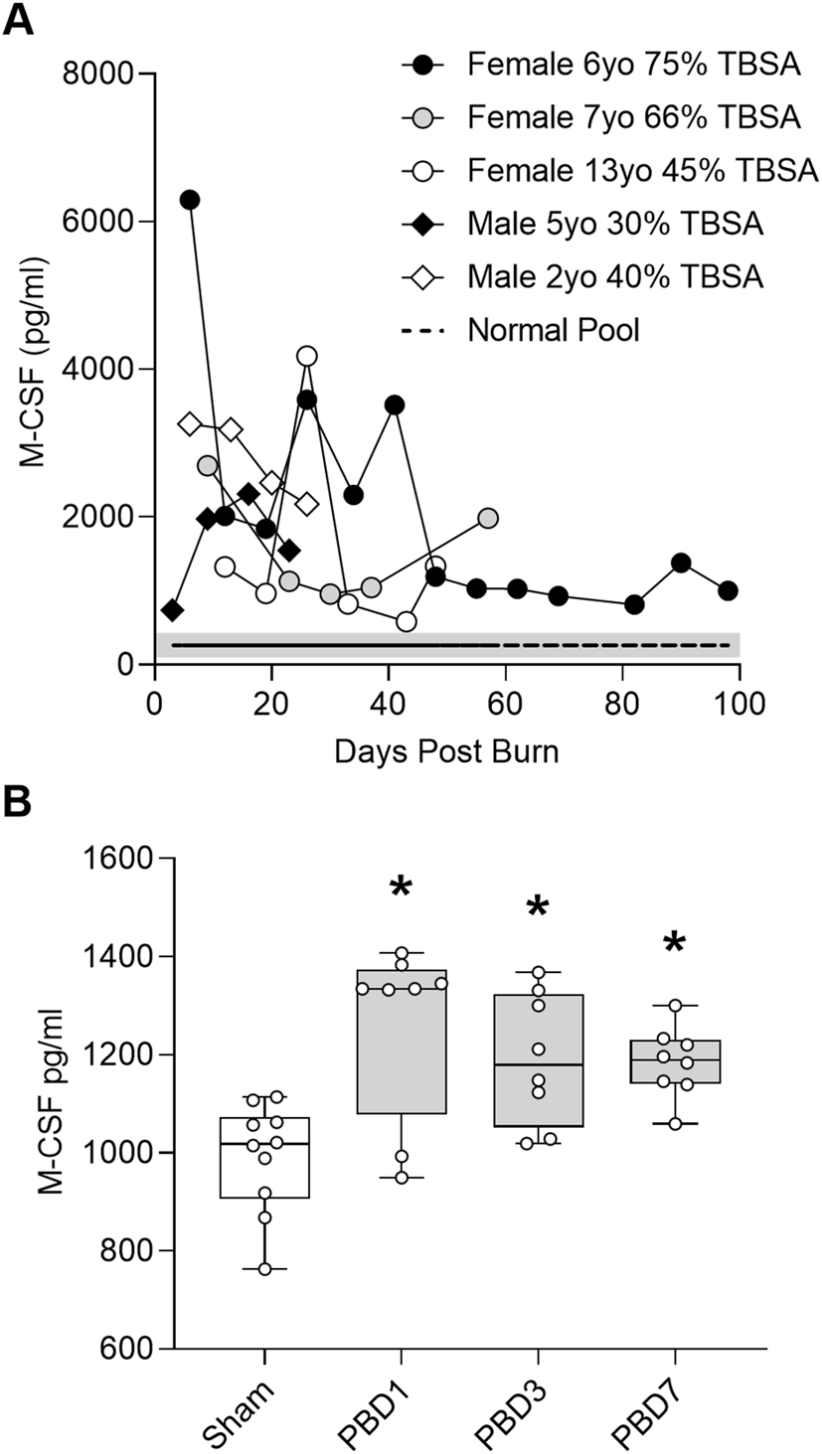
Burn injury induces M-CSF secretion in humans and mice. (**A**) Sample legend and plot showing M-CSF levels in burn patient plasma samples, n = 32. Shaded area represents the M-CSF ELISA kit manufacturer’s reported range (134–434 pg/ml) for healthy human plasma and the dashed line represents M-CSF levels measured in a pooled plasma sample from healthy humans. (**B**) Serum M-CSF levels in sham or burn injured mice on post burn day (PBD) 1, 3, and 7, n = 10 sham and n = 8 burn/day. Data shown as box plots with whiskers at min and max. *p < 0.05, ANOVA each post burn day versus sham.

To assess M-CSF secretion in burn patients we collected plasma discard from routine clinical testing. Stored samples were selected for measurement based on the availability of at least four serial weekly samples from the same patient to understand temporal secretion patterns. The samples included both genders, ages from two to thirteen years old, and burn sizes from 30 to 75% of total body surface area (TBSA). The manufacturer’s reported range for healthy humans was used as a reference and a pooled plasma sample from healthy volunteers confirmed the range. We found M-CSF levels were above the expected normal range in all burn plasma samples and generally followed a pattern in which secretion was elevated early and then waned over time (Fig. 1A). M-CSF levels were also elevated in mouse sera for at least one week following the 15% TBSA flame burn we administered (Fig. 1B). Together, this set of data indicated that burn injury initiates a durable increase of M-CSF secretion in both humans and mice.

### Effect of post burn M-CSF blockade on bone marrow cells

**Figure 2:**
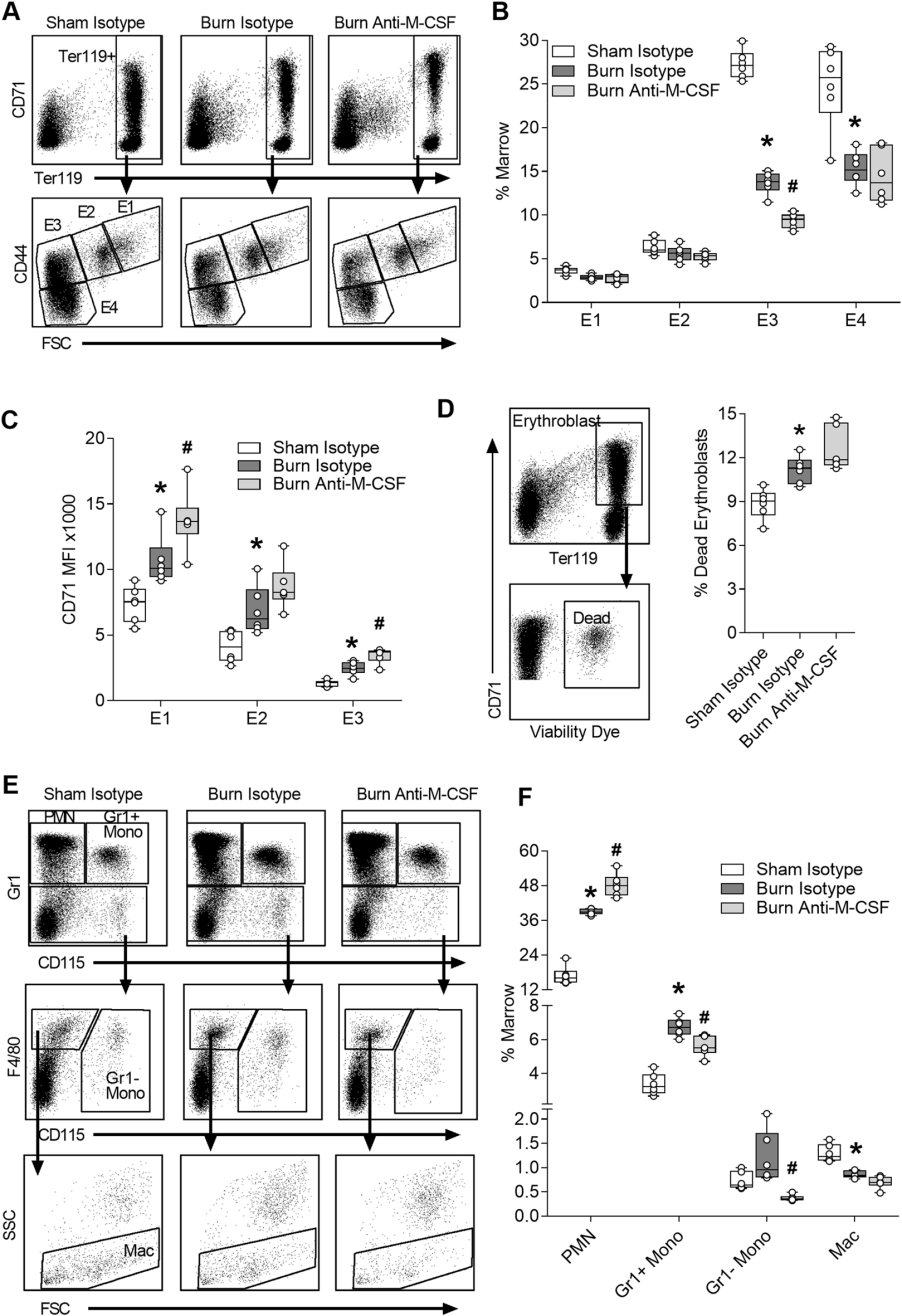
Effect of post burn M-CSF blockade on bone marrow cells. Bone marrow cells were harvested on day seven post injury from mice that received isotype or M-CSF neutralizing antibodies and analyzed using flow cytometry. (**A**) Gating of erythroid cells. (**B**) Erythroid cell populations plotted as % of marrow, n = 6/group. (**C**) CD71 MFI measured on the surface of erythroid populations E1 to E3, n = 6/group. (**D**) Gating and percentage of dead erythroblasts, n = 6/group. (**E**) Gating of myeloid cells after CD45+ pre-gate. (**F**) Myeloid populations plotted as % of marrow, n = 6/group. Data shown as box plots with whiskers at min and max. *p < 0.05 burn isotype versus sham isotype and ^#^*p* < 0.05 burn anti-M-CSF versus burn isotype, ANOVA.

To determine if M-CSF blockade improved medullary erythropoiesis in ACI mice, we administered isotype or M-CSF neutralizing antibodies immediately following injury and then daily for 6 days. Bone marrow cells were harvested on day seven and stained to phenotype erythroid and myeloid cells using flow cytometry. Erythroid cells were analyzed by gating on TER119+ cells and then CD44 and FSC to parse developing erythroid cells as previously described^32^ (Fig. 2A). TER119+ cells were significantly reduced in the bone marrow following burn injury (Sham Isotype, median 64%, range 55–69% vs. Burn Isotype, median 39%, range 34–42%; ANOVA *p* < 0.05) and tended to be further reduced in burn injured mice that received Anti-M-CSF (Burn Anti-M-CSF, median 33%, range 28–37%) compared to burn isotype-treated mice. The major reduction of TER119+ cells following burn injury is in late stage E3 and E4 populations (Fig. 2B), similar to our prior study^10^. The E3 population, a mix of orthochromatic erythroblasts and reticulocytes^32^, was reduced by 31% (Burn Isotype, median 13.8%, range 11.5–15% vs. Burn anti-M-CSF, median 9.5%, range 8.1–10.5%; ANOVA *p* < 0.05) in the burn anti-M-CSF group compared to the burn isotype group (Fig. 2B). CD71 surface staining intensity, a measure of transferrin receptor levels which can be elevated in iron restricted erythropoiesis^33–35^, was significantly increased in all erythroid cell populations isolated from burn isotype-treated mice compared to sham isotype-treated mice and further elevated in burn injured mice treated with M-CSF neutralizing antibodies (Fig. 2C). Erythroblast viability was measured by gating on CD71+TER119+ cells without prior exclusion of viability dye positive cells and then quantifying the percentage of dead erythroblasts as the proportion of erythroblasts that are viability dye positive. The percentage of dead erythroblasts was increased following burn injury, consistent with our prior study that linked this change to a G-CSF dependent impairment of EPO signaling^10^, and tended to be further increased in mice treated with M-CSF neutralizing antibodies (Fig. 2D). Bone marrow myeloid cells, pre-gated as CD45+, were parsed into neutrophils (PMN), Gr1 positive monocytes (Gr1+ Mono), Gr1 negative monocytes (Gr1− Mono) and macrophages (Mac) (Fig. 2E), as previously specified^36^. M-CSF blockade had divergent effects on myeloid populations, further increasing the bone marrow neutrophil contents while reducing the content of monocyte populations and tending to further reduce macrophages (Fig. 2F). To determine if the effects of M-CSF blockade were specific to burn injury, we used an identical neutralization strategy in mice that were not injured. M-CSF neutralization reduced bone marrow monocytes and macrophages in naïve mice but did not increase the bone marrow neutrophil contents (Supplementary Fig. 1). There were no significant changes in erythroid marrow composition (Supplementary Fig. 2A), erythroid CD71 levels (Supplementary Fig. 2B) or erythroblast viability (Supplementary Fig. 2C) in naïve mice subjected to M-CSF blockade. Together, these results indicated M-CSF blockade had unique effects in burn injured animals that further impaired measures of erythropoiesis and increased neutrophil counts.

### Effect of post burn M-CSF blockade on spleen cells

**Figure 3:**
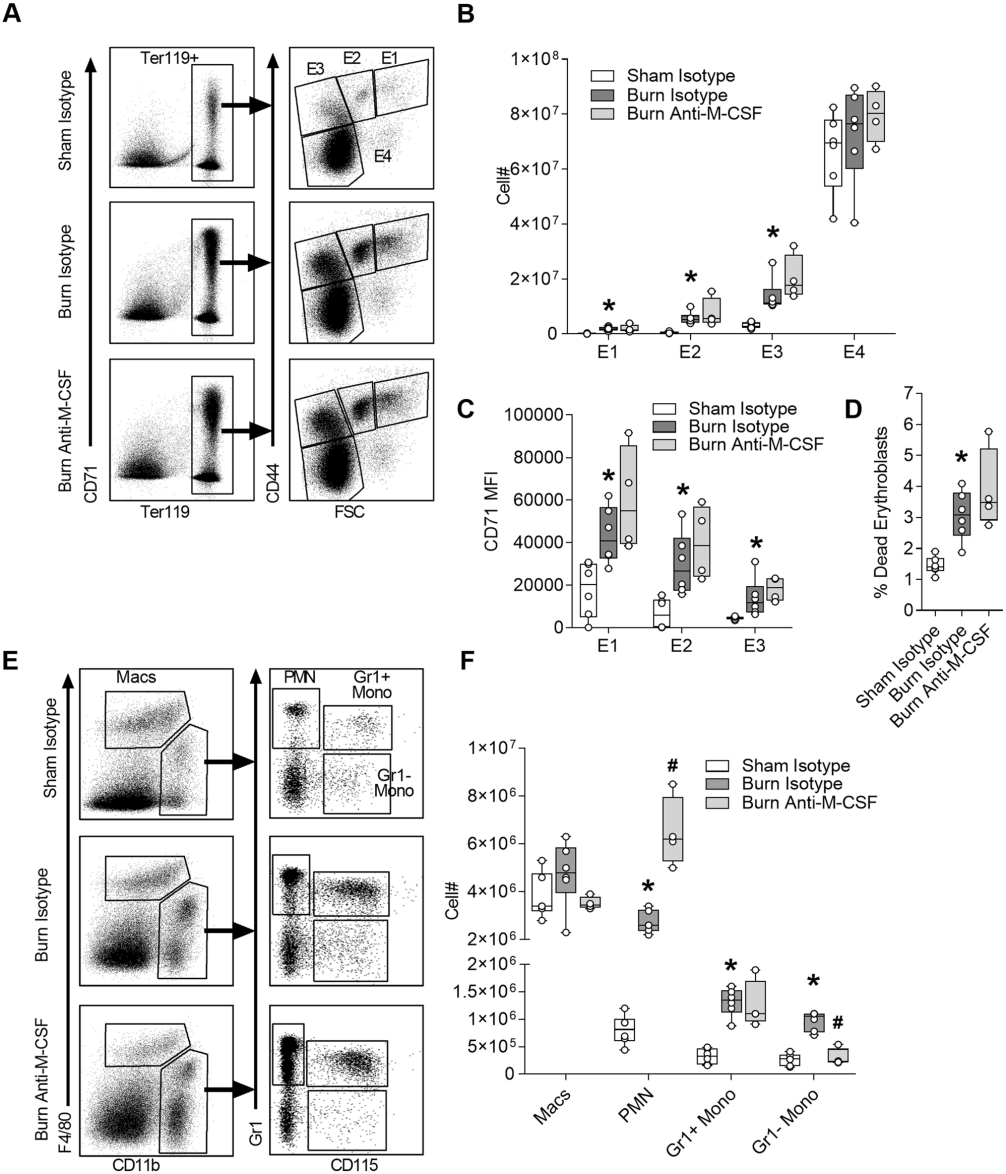
Effect of post burn M-CSF blockade on spleen cells. Spleen cells were harvested on day seven post injury from mice that received isotype or M-CSF neutralizing antibodies and analyzed using flow cytometry. (**A**) Gating of erythroid cells. (**B**) Total erythroid cell counts per spleen, n = 4–6/group. (**C**) CD71 MFI measured on the surface of erythroid populations E1 to E3, n = 4–6/group. (**D**) Percentage of dead erythroblasts, n = 4–6/group. (**E**) Gating of myeloid cells after selecting the CD45+ population. (**F**) Total myeloid cell counts per spleen, n = 4–6/group. Data shown as box plots with whiskers at min and max. *p < 0.05 burn isotype versus sham isotype and ^#^*p* < 0.05 burn anti-M-CSF versus burn isotype, ANOVA.

Extramedullary hematopoiesis is well-known to be initiated in the spleens of mice subjected to burn injury and limits the severity of anemia^37,38^, so we assessed the role of M-CSF in this response using the neutralization strategy and analysis on day seven post injury. Erythroid cell counts were determined by gating on the TER119+ population and then parsed based on increasing maturity using CD44 versus FSC parameters (Fig. 3A). All early erythroid populations (E1 to E3) were significantly increased following burn injury irrespective of antibody treatment, consistent with burn injury inducing emergency erythropoiesis, while the large E4 population that includes a disproportionate number of circulating cells was not (Fig. 3B). CD71 surface staining intensity on splenic erythroid populations (Fig. 3C) and the proportion of dead erythroblasts (Fig. 3D) were also increased following burn injury irrespective of antibody treatment. Analysis of splenic myeloid populations was performed using F4/80 versus CD11b parameters to identify macrophages (Macs) and then parsing other CD11b+ populations using Gr1 versus CD115 parameters into neutrophils (PMN), Gr1+ monocytes (Gr1+ Mono) and Gr1− monocytes (Gr1− Mono) (Fig. 3E). Similar to the bone marrow, neutrophils were increased in the spleen following burn injury and further elevated in burn injured mice subjected to M-CSF blockade (Fig. 3F). Macrophages and monocytes followed a similar pattern of increase after burn injury, but a statistical increase was only apparent in monocytes and a reversal with M-CSF blockade was limited to the Gr1− monocyte population (Fig. 3F). Together, these results confirmed extramedullary hematopoiesis is initiated by burn injury and indicated the primary effects of post burn M-CSF blockade were increased neutrophil counts and attenuation of the Gr1- monocyte increase.

### Effect of post burn M-CSF blockade on circulating blood cells

To determine if the changes in tissues were reflected peripherally, we used the neutralization strategy and collected blood on day seven for analysis. Neutrophils and platelets were elevated 7 days after burn injury, as in our prior studies that utilized a different hematology system^10,11^, and M-CSF blockade further increased post burn neutrophil counts (Table 1). Surprisingly, post burn M-CSF blockade increased red blood cell counts but hemoglobin and hematocrit were not significantly improved, a discrepancy that could be explained by a reduction in mean corpuscular volume (MCV) (Table 1). The increased red blood cell count was not associated with an increase of reticulocytes and although the immature reticulocyte fraction (IRF-M + H) indicated some recent erythropoietic activity, consistent with the induction of splenic erythropoiesis, the IRF-M + H always tended to be lower in burn injured mice subjected to M-CSF blockade (Table 1). M-CSF blockade also increased the proportion of reticulocytes with low cellular hemoglobin content (Low CH Retic) and tended to further increase the proportion of reticulocytes with a low hemoglobin concentration (Hypo Retic) that was apparent in the burn isotype group (Table 1). In some experiments we collected bone marrow and blood from the same mice and found increased CD71 staining intensity on bone marrow erythroid cells was well correlated with the increase of hypochromic reticulocytes in blood (Supplementary Fig. 3). Notably, prior reports identified a similar relationship between increased CD71 staining intensity on circulating reticulocytes and hypochromic reticulocytes that reflected iron deficiency at the cellular level^34,35^. We used an identical neutralization strategy in non-injured mice to determine if the effects of M-CSF blockade were specific to burn injury. M-CSF blockade in naïve mice did not alter mature blood cell parameters (Supplementary Fig. 4A) or reticulocyte parameters except for a slight but statistically significant increase in the proportion of reticulocytes with low cellular hemoglobin (Supplementary Fig. 4B). Overall, these data confirmed post burn M-CSF blockade systemically increases neutrophil counts and further limits erythroid cells access to iron.

**Table 1:** Effect of M-CSF neutralization on blood cells.

Parameter, units	Sham + isotype (n = 6)	Burn + isotype (n = 6)	Burn + anti-M-CSF (n = 6)
Neutrophils, K/µl	0.15 ± 0.03	0.68 ± 0.41*	1.23 ± 0.44^**#**^
Monocytes, K/µl	0.03 ± 0.03	0.05 ± 0.03	0.03 ± 0.01
Lymphocytes, K/µl	1.03 ± 0.29	1.06 ± 0.54	1.21 ± 0.50
Platelets, K/µl	826 ± 181	1194 ± 116*	1307 ± 120
Red cells, M/µl	8.64 ± 0.30	8.18 ± 0.21*	8.58 ± 0.32^**#**^
Hemoglobin, g/dL	14.10 ± 0.44	12.93 ± 0.12*	13.30 ± 0.42
Hematocrit, %	45.07 ± 1.30	42.22 ± 1.28*	42.95 ± 1.83
MCV, fL	52.17 ± 0.76	51.65 ± 1.24	50.08 ± 0.47^**#**^
Reticulocytes, K/µl	281 ± 22	318 ± 33	334 ± 65
IRF-M + H, %	58.0 ± 2.4	69.5 ± 2.8*	66.2 ± 3.7
Low CH retic, %	28.2 ± 3.2	31.5 ± 10.3	46.7 ± 5.7^**#**^
Hypo retic, %	9.9 ± 4.7	21.8 ± 9.6*	28.4 ± 3.8

Parameters determined on an automated hematology system are presented as mean ± SD.

Significance, ANOVA *p* < 0.05 is indicated by * for burn isotype versus sham isotype and ^**#**^for burn anti-M-CSF versus burn isotype.

### Post burn M-CSF blockade augments secretion of inflammatory mediators and increases tissue iron sequestration

**Figure 4:**
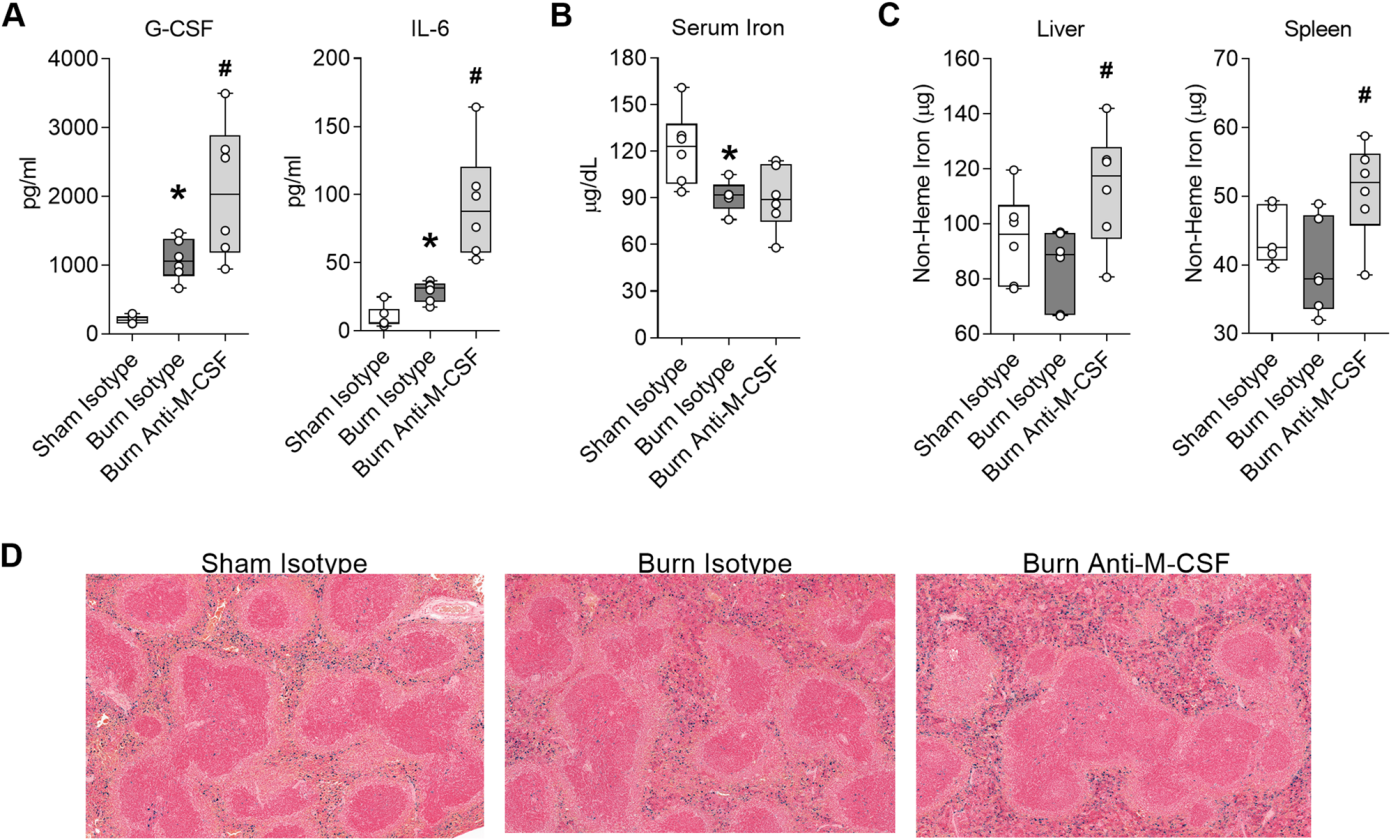
Post burn M-CSF blockade augments secretion of inflammatory mediators and increases tissue iron sequestration. Blood and tissues were collected on day seven post injury from mice that received isotype or M-CSF neutralizing antibodies. (**A**) Serum cytokine levels, n = 6/group. (**B**) Serum iron, n = 5–6/group (**C**) Total non-heme iron levels in liver and spleen, n = 5–6/group. (**D**) Representative Prussian blue stained spleen tissue, 10x. Data in panels (**A**) through (**C**) shown as box plots with whiskers at min and max. *p < 0.05 burn isotype versus sham isotype and ^#^*p* < 0.05 burn anti-M-CSF versus burn isotype, ANOVA.

Post burn M-CSF blockade led to a further increase of neutrophils in tissues and circulation which could portend an exaggerated inflammatory response. We quantified G-CSF and IL-6 to assess the effect of post burn M-CSF blockade on inflammation because these factors remained elevated on post burn day seven in our prior studies, albeit levels were highest at earlier post burn time points^10,11^. As expected, both factors were still elevated in isotype-treated burn injured mice 1 week after the insult (Fig. 4A). Comparisons of the burn isotype and burn anti-M-CSF groups revealed M-CSF blockade augmented G-CSF levels by 92% (Burn Isotype, median 1057 pg/ml, range 666–1467 pg/ml vs. Burn Anti-M-CSF, median 2033 pg/ml, range 948–3496 pg/ml; ANOVA *p* < 0.05) and IL-6 levels by 175% (Burn Isotype, median 32 pg/ml, range 18–37 pg/ml vs. Burn Anti-M-CSF, median 88 pg/ml, range 52–164 pg/ml; ANOVA *p* < 0.05) (Fig. 4A). Since post burn M-CSF blockade worsened reticulocyte indices of iron deficiency, we also assessed serum and tissue iron levels. Serum iron levels were reduced one week after burn injury regardless of antibody treatment (Fig. 4B), but post burn M-CSF blockade did increase total non-heme iron levels by 33% in the liver (Burn Isotype, median 89 µg, range 67–97 µg vs. Burn Anti-M-CSF, median 118 µg, range 81–142 µg; ANOVA *p* < 0.05) and 37% in the spleen (Burn Isotype, median 38 µg, range 32–49 µg vs. Burn Anti-M-CSF, median 52 µg, range 39–59 µg; ANOVA *p* < 0.05) (Fig. 4C). The increased tissue iron sequestration caused by post burn M-CSF blockade was visually obvious in splenic sections stained with Prussian blue (Fig. 4D). Together, these results indicated M-CSF blockade led to exaggerated inflammatory cytokine secretion and increased tissue iron sequestration in burn injured mice.

### M-CSF secretion supports the induction of an iron recycling program in the liver and spleen following burn injury

**Figure 5:**
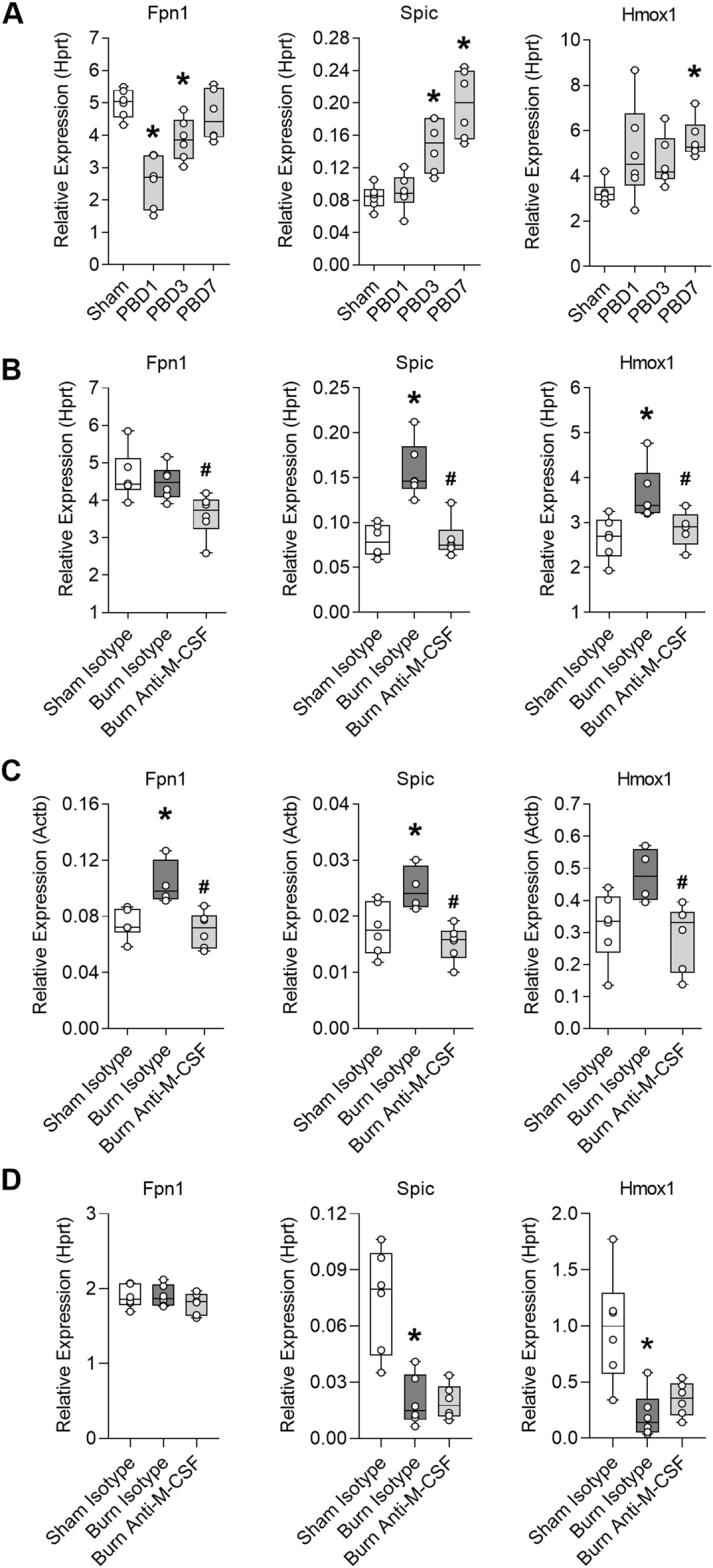
M-CSF secretion supports the induction of an iron recycling program in the liver and spleen following burn injury. (**A**) Hepatic gene expression in sham or burn injured mice on post burn day (PBD) 1, 3, and 7, n = 6/group. Data shown as box plots with whiskers at min and max. *p < 0.05, ANOVA each post burn day versus sham. Liver, spleen, and bone marrow were harvested on day seven post injury from mice that received isotype or M-CSF neutralizing antibodies to assess gene expression. (**B**) Liver expression, n = 6/group. (**C**) Spleen expression, n = 4–6/group. (**D**) Bone marrow expression, n = 6/group. Data shown as box plots with whiskers at min and max. *p < 0.05 burn isotype versus sham isotype and ^#^*p* < 0.05 burn anti-M-CSF versus burn isotype, ANOVA.

The liver is a key site involved in M-CSF dependent recycling of damaged erythrocytes^16^, so we assessed the expression pattern of genes involved in the hepatic iron recycling program, ferroportin (Fpn1), Spi-C transcription factor (Spic), and heme oxygenase 1 (Hmox1) over one week post injury. We found Fpn1 expression was reduced by nearly 50% on PBD1 and then tended to recover by PBD7 (Fig. 5A). Spic and Hmox1 increased over time following burn injury, with Spic increased 150% and Hmox1 increased 66% in PBD7 livers compared to livers harvested from sham injured mice (Fig. 5A). We noted the recovery of hepatic Fpn1 expression was temporally related to elevation of Spic and Hmox1 which suggested the response may be an adaptation to increased erythrocyte destruction that is supported by M-CSF^16,17^. To determine if the iron recycling transcriptional program induced by burn injury was M-CSF dependent and restricted to the liver, we used the neutralization strategy to assess gene expression in the liver, spleen, and bone marrow on post injury day seven. Hepatic Fpn1 expression was similar in isotype-treated sham or burn injured mice, consistent with the recovery of Fpn1 expression by PBD7 shown in Fig. 5A, but the recovery of Fpn1 in burn injured mice was attenuated by M-CSF blockade (Fig. 5B). Hepatic Spic and Hmox1 expression were elevated by burn injury, as expected from the time course experiments, and the elevations were M-CSF dependent (Fig. 5B). The iron recycling transcriptional response was also evident in the spleen following burn injury and was sensitive to M-CSF blockade (Fig. 5C). In the bone marrow, Fpn1 expression was not altered by burn injury or M-CSF blockade and the expression of Spic and Hmox1 were markedly reduced irrespective of antibody treatment (Fig. 5D). Together, these data indicated burn injury induced an M-CSF dependent iron recycling program in the liver and spleen that may serve as an iron homeostasis response to increased erythrocyte destruction.

### M-CSF secretion supports monocyte differentiation and expansion of iron recycling macrophages in the liver following burn injury

**Figure 6:**
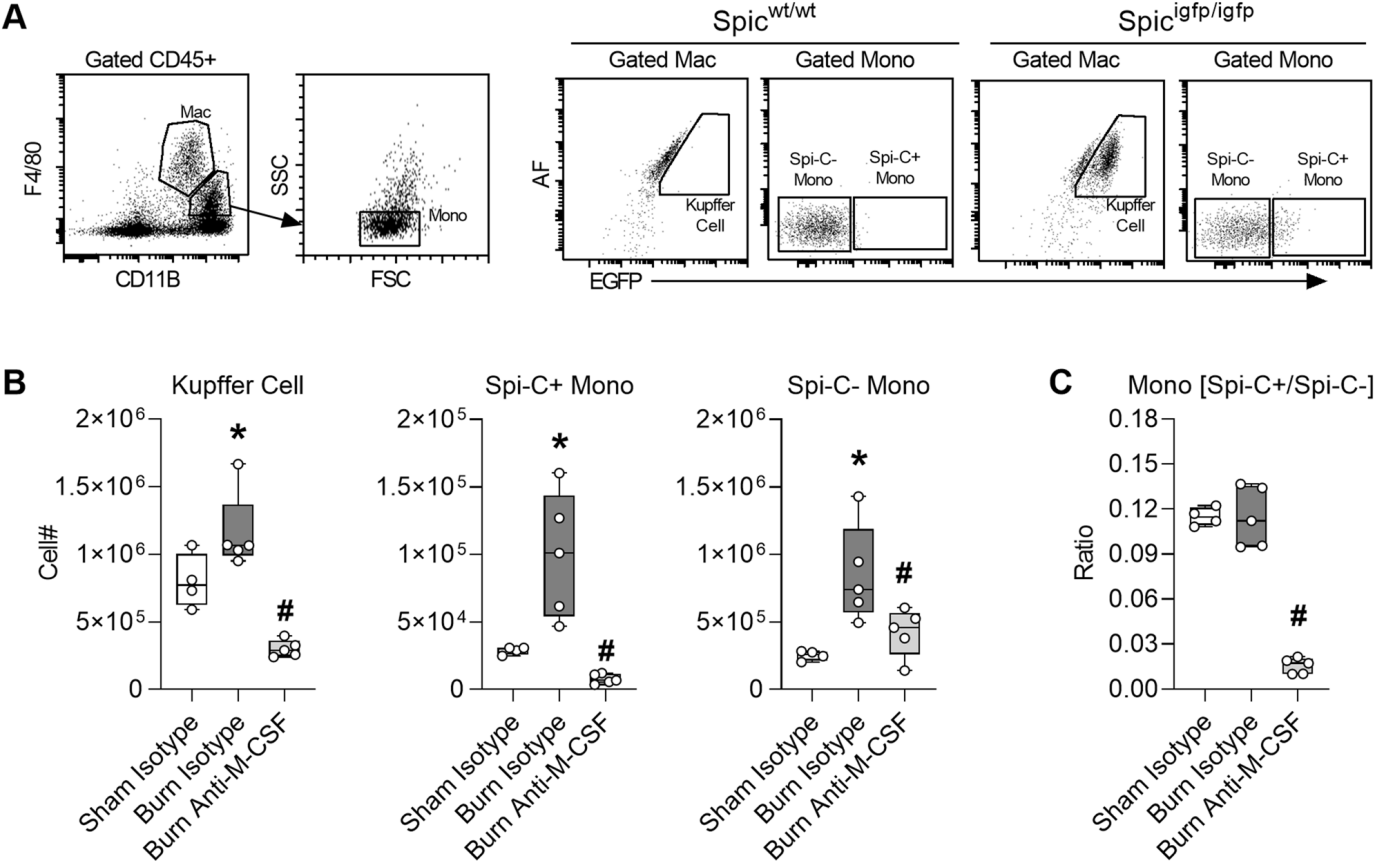
M-CSF secretion supports monocyte differentiation and expansion of iron recycling macrophages in the liver following burn injury. Liver cells were isolated on day seven post injury from Spi-C reporter mice that received isotype or M-CSF neutralizing antibodies. (**A**) Gating strategy. (**B**) Cell counts, n = 4–5/group. (**C**) Ratio of Spi-C+ monocytes/Spi-C− monocytes, n = 4–5/group. Data shown as box plots with whiskers at min and max. *p < 0.05 burn isotype versus sham isotype and ^#^*p* < 0.05 burn anti-M-CSF versus burn isotype, ANOVA.

Spi-C has been identified as a lineage determining transcription factor expressed in monocytes that differentiate into Kupffer cells and in Kupffer cells^39^, so we utilized Spi-C reporter mice^17^ to interrogate differentiation of monocytes into Kupffer cells in ACI mice subjected to M-CSF blockade. Cells isolated from the liver on post injury day seven were assessed by flow cytometry using standard markers and autofluorescence (AF) to quantify Kupffer cells (F4/80++, CD11b+, AF high, Spi-C+) and monocytes (F4/80+, CD11b++, FSC/SSC low, AF low) as Spi-C positive or Spi-C negative (Fig. 6A). The post burn expansion of Kupffer cells, Spi-C+ monocytes, and to some extent Spi-C− monocytes in the liver were M-CSF dependent (Fig. 6B). Comparing the ratio of Spi-C+/Spi-C− monocytes in each group indicates these monocyte populations increase in tandem following burn injury but the expansion of Spi-C+ monocytes is most reliant on M-CSF secretion (Fig. 6C). Together, these data indicate post burn M-CSF secretion plays a key role in the expansion and differentiation of Spi-C+ monocytes into iron recycling macrophages within the liver.

### Exacerbation of iron restricted erythropoiesis caused by post burn M-CSF blockade is at least in part IL-6 dependent and associated with induction of hepcidin

**Figure 7:**
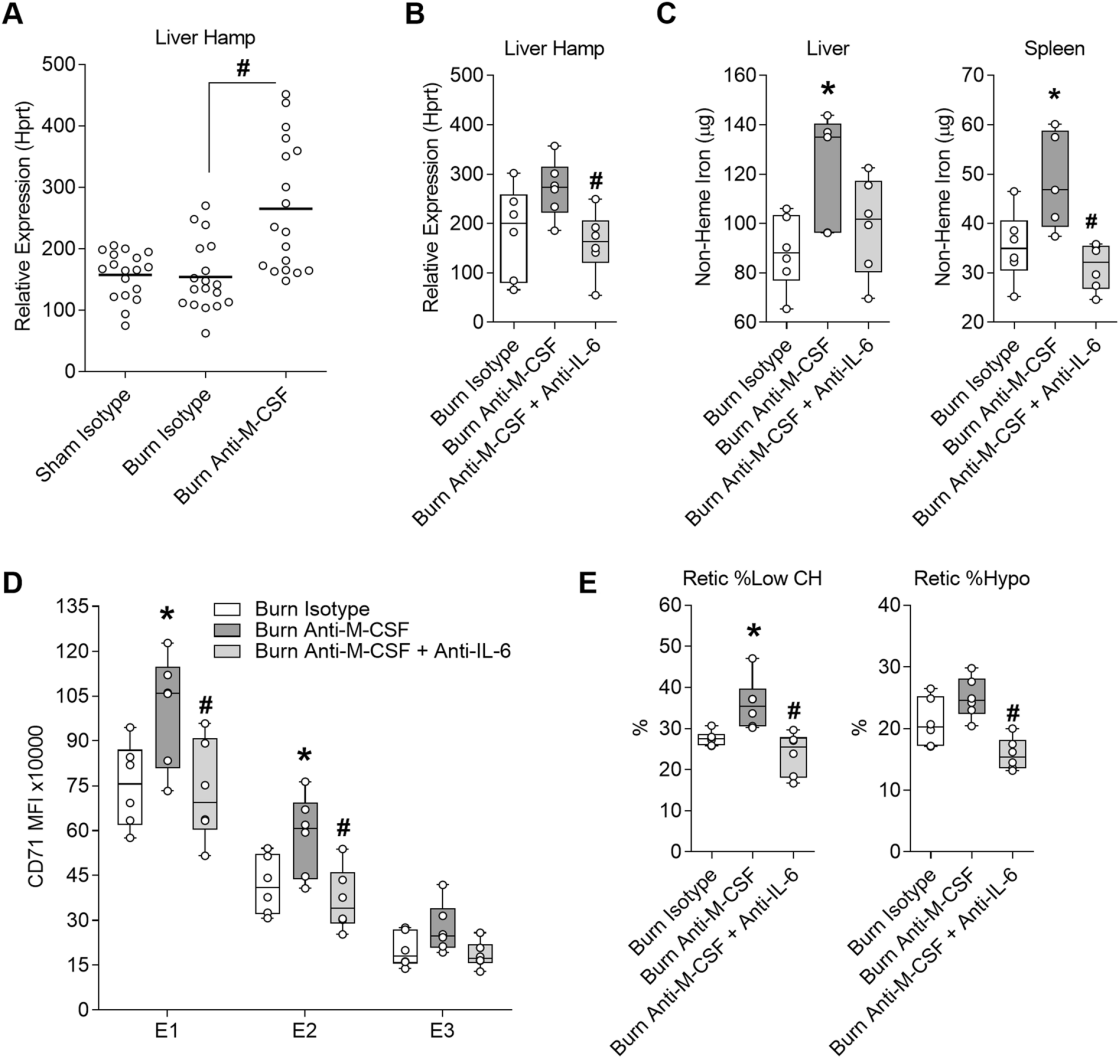
Exacerbation of iron restricted erythropoiesis caused by post burn M-CSF blockade is at least in part IL-6 dependent and associated with induction of hepcidin. Samples were harvested on day seven post injury from mice that received isotype or M-CSF neutralizing antibodies or from post burn day seven mice that received isotype, M-CSF neutralizing, or a combination of M-CSF and IL-6 neutralizing antibodies. (**A**) Pooled analysis of liver hepcidin expression relative to Hprt from three independent experiments for a total of n = 18/group. (**B**) Liver hepcidin expression relative to Hprt for n = 6/group. (**C**) Total non-heme iron levels in liver and spleen n = 5–6/group. (**D**) CD71 MFI of bone marrow erythroid populations E1 to E3 for n = 6/group. (**E**) ADVIA reticulocyte iron indices for n = 6/group. Panel (**A**) shown as dot plot with line at mean, *p < 0.05 sham isotype versus burn isotype and ^#^*p* < 0.05 burn isotype versus burn anti-M-CSF, ANOVA. All other panels shown as box plots with whiskers at min and max, *p < 0.05 burn isotype versus burn anti-M-CSF and ^#^*p* < 0.05 burn anti-M-CSF versus burn anti-M-CSF+ anti-IL-6.

Ferroportin is the only known iron exporter in mammals and the level of ferroportin on the membrane of cells involved in iron absorption and recycling is a primary determinant of iron availability. Ferroportin is regulated transcriptionally, post-transcriptionally through mRNA stability, and through protein turnover^40^. Hepcidin is the most well-known post-translational regulator of ferroportin that acts by degrading ferroportin on the surface of cells and preventing release of iron from tissues into plasma^41^. Hepcidin is predominantly expressed in the liver and regulated by several inputs; inflammation, iron sensing, and hormones produced by erythroblasts^42^, among which synergies between iron signaling and IL-6 signaling are required for robust hepcidin induction^43^. We assessed hepcidin expression in the livers of mice over one week post burn, but we could only identify a trend towards increased hepcidin (Hamp) expression on post burn day one (Supplementary Fig. 5) suggesting hepcidin may be induced early after burn injury when IL-6 levels are highly elevated^10,11^. Since post burn M-CSF blockade augmented IL-6 secretion, we evaluated liver Hamp expression in three independent M-CSF neutralization experiments on post burn day seven. Hamp expression was increased in burn injured mice that received M-CSF neutralizing antibodies compared to burn isotype-treated mice after pooling the data (Fig. 7A). To determine if IL-6 modulated Hamp expression and iron related changes in burn injured mice treated M-CSF neutralizing antibodies, we used a dual M-CSF and IL-6 neutralization strategy. Mice were harvested on post burn day seven for quantification of liver Hamp expression, determination of iron stores in liver and spleen, CD71 surface staining intensity on bone marrow erythroid cells, and determination of reticulocyte iron indices. Combined neutralization of M-CSF and IL-6 reduced liver Hamp expression compared to mice that only received M-CSF neutralizing antibodies (Fig. 7B). Consistent with the reduction of Hamp expression, combined neutralization of M-CSF and IL-6 reduced tissue iron sequestration, albeit statistical reduction was limited to the spleen (Fig. 7C). Combined neutralization of M-CSF and IL-6 also reduced the elevation of TFR1/CD71 on bone marrow erythroid cells (Fig. 7D) and the proportion of circulating reticulocytes with low hemoglobin levels (Fig. 7E) compared to burn injured mice that received M-CSF neutralizing antibodies alone. Together, these data indicate post burn M-CSF blockade exacerbates iron restricted erythropoiesis at least in part through augmenting IL-6 secretion which induces hepcidin.

## Discussion

Iron recycling macrophages are the principal source of iron used for erythropoiesis and we found M-CSF blockade further impaired the access of erythroid cells to iron in ACI mice. Post burn M-CSF blockade led to further deterioration in reticulocyte indices of iron deficiency, % Low CH and % Hypo, and augmented a burn induced elevation of erythroid transferrin receptor which can be a measure of iron availability at the cellular level^33–35^. Iron restricted erythropoiesis caused by post burn M-CSF blockade was associated with increased tissue iron sequestration and arrest of an iron recycling transcriptional program in ACI mice that was required for recovery of hepatic ferroportin gene expression and increased splenic ferroportin gene expression. The hepatic transcriptional program reflected an M-CSF dependent expansion of Spi-C+ monocytes and differentiation into Kupffer cells following burn injury, reminiscent of M-CSF dependent homeostatic adaptation to increased heme burden that serves to restore iron homeostasis^16^. Insufficient macrophage iron recycling function commonly leads to accumulation of iron in tissues. Non-heme iron increases in Nramp1−/− mice which do not efficiently recycle iron from damaged red blood cells^44^, in myeloid specific Fpn1−/− mice due to impaired mobilization from macrophages^45^, in Hmox1−/− mice due to heme toxicity that leads to broad depletion of iron recycling macrophages^21,46^, and Spic−/− mice due to loss of red pulp macrophages^23^. Further, we found the exacerbation of iron restricted erythropoiesis caused by post burn M-CSF neutralization was associated with augmented secretion of G-CSF and IL-6. Importantly, elevated hepcidin expression, tissue iron sequestration, and iron restricted erythropoiesis caused by post burn M-CSF blockade were mitigated by combined M-CSF and IL-6 blockade. Since IL-6 promotes tissue iron sequestration through induction of hepcidin^47^ and we could only detect a significant induction of hepcidin in M-CSF neutralized ACI mice, our results suggest the augmented IL-6 secretion may have been required to overcome regulatory mechanisms such as low iron levels or erythropoietic hormones which attenuate hepcidin expression^42^. The current study does not identify the precise mechanism that led to augmented IL-6 secretion but exaggerated inflammatory responses have been linked to impaired macrophage iron recycling function. Myeloid specific Hmox1, Spic and Fpn1 deficiency all lead to excess production of inflammatory cytokines by macrophages^18,19,45^. In the studies that assessed IL-6, Spi-C attenuated IL-6 secretion through an interaction with IRF5 that disrupted NF-ϰB complex formation^18^ and increased IL-6 secretion by Fpn1 deficient cells was associated with an increase of intracellular iron that was suspected to enhance NF-ϰB signaling^45^. Together, these results suggest M-CSF supports a homeostatic iron recycling program that promotes the expansion and/or recovery of ferroportin expressing macrophages and a non-inflammatory tone in macrophages that limits IL-6/hepcidin mediated ferroportin protein degradation following burn injury.

Post burn M-CSF blockade led to a further impairment of medullary erythropoiesis that was most apparent at or near the orthochromatic erythroblast stage. The further reduction of erythroid cellularity caused by post burn M-CSF blockade may have occurred due to augmented G-CSF secretion since we previously found G-CSF impairs medullary erythropoiesis in ACI mice^10^ or because iron restriction impairs late stage differentiation and enucleation^48,49^. The augmented G-CSF secretion likely contributed to the systemic neutrophil increase we measured in burn injured mice subjected to M-CSF blockade because we previously determined the post burn neutrophil increase is G-CSF dependent^11^. As in most anemia models, compensatory extramedullary erythropoiesis was induced by burn injury, but the response was very similar in burn injured mice that received M-CSF neutralizing antibodies. The induction of splenic erythropoiesis was associated with a small increase of immature reticulocytes in the blood of burn injured mice regardless of antibody treatment, but the increase was always attenuated to some extent in burn injured mice that received M-CSF neutralizing antibodies compared to burn injured mice that received isotype antibodies. Although M-CSF blockade further impaired erythropoiesis in the bone marrow and did not increase erythropoiesis in the spleen, we observed an increase in circulating red blood cell counts in M-CSF neutralized ACI mice that occurred without improving hemoglobin or hematocrit and was associated with a reduction of MCV. We did not observe similar changes in the parameters when naïve mice were subjected to M-CSF blockade over a one week period, but a prior report indicates extended M-CSF receptor blockade led to a small increase of circulating red blood cells and a reduction of MCV in healthy mice^50^. Given that M-CSF blockade arrested the post burn induction of a macrophage iron recycling program in the liver and spleen, the increase of microcytic red blood cells in the absence of improved erythropoiesis may have occurred due to modification of erythrocyte clearance function.

Prior studies indicate myeloid biased lineage commitment plays a role in the impairment of bone marrow erythropoiesis following burn injury^12–15^ and propose increased expression of M-CSF receptors in LSK cells driven by adrenergic receptor signaling as a possible mechanism of myeloid bias^14,15^. The role of M-CSF receptor or M-CSF in post burn ACI were never explicitly tested, but the concept of M-CSF driven myeloid bias is consistent with a role for M-CSF instruction of lineage fate in hematopoietic stem cells^51^. Based on the prior studies and the increased M-CSF secretion we measured in burn injured humans and mice, we directly tested the role of M-CSF secretion in post burn ACI for the first time. The results of our study are more consistent with the essential role of M-CSF/M-CSF receptor signaling at a later stage that supports maturation and replacement of resident-type monocytes and tissue macrophages^52–54^. In summary, the M-CSF/M-CSF receptor signaling axis plays a key role in post burn ACI that supports iron recycling macrophages and erythroid cells access to iron. We speculate this system functions as an adaptive response to increased erythrocyte damage following burn injury that is meant to restore iron homeostasis. A more complete understanding of the mechanism could reveal therapeutic approaches that promote a counter inflammatory iron recycling program and resolution of ACI.

## Methods

### Animal model

All experiments were performed according to the protocol approved by the Institutional Care and Use Committee at the University of Cincinnati (IACUC Protocol# 06-06-16-01). All methods were performed following relevant guidelines and regulations in the approved protocol and are reported according to ARRIVE guidelines. Eight-week-old female C57BL/6J mice were purchased from The Jackson Laboratory and maintained on irradiated NIH-07 Mouse/Rat Diet containing 350 mg/kg iron (Envigo, Madison, WI) for at least one week prior to experiments. B6.129S6(C)-*Spic*^*tm2.1Kmm*^/J and C57BL/6J control mice were raised in house on the same diet. Mice were subjected to a 15% total body surface area (TBSA) full-thickness flame burn injury as previously described^11^. In brief, mice were anesthetized with isoflurane and covered with a flame-resistant template exposing the shaved dorsal skin. The target area was saturated with 0.5 ml of absolute ethanol and ignited for 10 s in the burn group or allowed to evaporate without ignition in the sham group. Immediately after the procedure, all mice received 0.5 ml of saline for volume resuscitation via the intraperitoneal (i.p.) route. Mice were sacrificed by i.p. injection of Euthasol (Virbac, Fort Worth, TX).

### Neutralization studies

Mice were administered 200 µg of anti-CSF1 clone 5A1 (catalog no. BE0204; Bio X Cell) or Rat IgG1 clone HRPN (catalog no. BE0088; Bio X Cell) isotype control by the i.p. route in a 100 µl volume of PBS immediately post injury and then daily for 6 days. For combined M-CSF and IL-6 neutralization, mice were administered 400 µg of isotype, 200 µg of isotype and 200 µg of anti-CSF1, or 200 µg anti-CSF1 and 200 µg of anti-IL-6 clone MP5-20F3 (catalog no. BE0046; Bio X Cell) by the i.p. route in a 200 µl volume of PBS immediately post injury and then daily for 6 days.

### Blood analysis

Blood was obtained from mice by cardiac puncture and placed in EDTA coated tubes (Microvette 500 K3E, Sarstedt, Germany) or serum separator tubes (Microvette 500 Z-Gel, Sarstedt). Cell counts were performed on an ADVIA 2120i Hematology System (Siemens Healthcare) within two hours of collection. Serum was stored at – 80 °C until quantification of G-CSF and IL-6 using Milliplex MAP kits (Millipore, Billerica, MA), quantification of M-CSF using the Mouse M-CSF Quantikine ELISA Kit MMC00 (R&D Systems, Minneapolis, MN), or determination of serum iron levels using a QuantiChrom Iron Assay Kit (BioAssay Systems, Hayward, CA). All human experimental protocols were approved by the University of Cincinnati Institutional Review Board, Study Approval #2014-8661. Informed consent for collection of human plasma was waived by the University of Cincinnati Institutional Review Board because the samples were obtained as laboratory discard without individual patient identifiers (Study Approval #2014-8661). The study was carried out in accordance with all relevant guidelines, regulations, and the Declaration of Helsinki. Excess pediatric burn plasma from routine clinical testing was stored daily at − 80 °C, then thawed one time to aliquot into appropriate assay volumes and a second time immediately prior to assay using the Human M-CSF Quantikine ELISA Kit DMC00B (R&D Systems, Minneapolis, MN). A pool of healthy adult human plasma from both genders served as a control to confirm the normal range specified by the ELISA kit.

### Isolation of cells and flow cytometry

Bone marrow cells were flushed from each femur with 1.5 ml of HBSS and spleen cells were isolated by applying gentle circular pressure to the tissue resting atop a 70 µm filter with a syringe plug while continuously rinsing with HBSS. Liver cells were isolated by dissociation using a gentleMACS (Miltenyi Biotec, Auburn, CA). Liver dissociation buffer contained 5 ml HBSS, 2.5 U/ml Dispase (catalog no. 07913; Stemcell Technologies), 500 U/ml Type IV collagenase (catalog no. LS004188; Worthington Biochemical), and 100 U/ml DNase I (catalog no. LS002139; Worthington Biochemical). When red blood lysis is specified, cells were suspended in RBC Lysis Buffer (catalog no. 00-4300-54; eBioscience) according to manufacturer’s instructions. Isolated cells were incubated in viability dye eFluor 780 (eBioscience) and then washed in ice-cold FACS buffer (DBPS, 1% BSA and 0.1% sodium azide). Non-specific binding was blocked with rat serum and Mouse Fc Block (BD Biosciences) for 10 min on ice prior to staining with fluorescent labeled antibodies for 30 min. The antibody panel for bone marrow and spleen erythroid cell analysis included TER-119 (clone TER-119), CD71 (clone R17217), CD44 (clone IM7) and CD45 (clone 30-F11), all from eBioscience. The antibody panel for bone marrow myeloid cell analysis included TER-119 (clone TER-119), Gr-1 (clone RB6-8C5), and CD45 (clone 30-F11), from eBioscience as well as CD115 (clone T38-320) and F4/80 (clone T45-2342) from BD Biosciences. The same myeloid panel was used for spleen cells, except CD11b (clone M1/70) from eBioscience was substituted for TER-119. The panel for analysis of livers from Spi-C reporter mice included F4/80 (clone T45-2342) from BD Biosciences as well as CD45 (clone 30-F11) and CD11b (clone M1/70) from eBioscience. Cells were washed three times with ice-cold FACS buffer prior to performing flow cytometry on an LSRII or LSRFortessa from BD Biosciences or an Attune (Thermo Fisher). Data were analyzed using FCS Express 5 (DeNovo Software).

### Gene expression

Total RNA was isolated using RNAzol^®^ RT (Molecular Research Center, Cincinnati, OH) and converted to cDNA with a High-Capacity cDNA Reverse Transcription Kit (Applied Biosystems, Foster City, CA). Power SYBR^®^ Green PCR Master Mix (Applied Biosystems, Foster City, CA) was used to amplify DNA fragments through 40 cycles at 95 °C for 15 s followed by 60 °C for 1 min. Primers are listed in Supplementary Table 1. Expression was determined relative to the housekeeping genes hypoxanthine guanine phosphoribosyl transferase (Hprt) or actin beta (Actb) as specified in plots.

### Iron assay and staining

Non-heme iron in tissues was quantified according to the method of Torrance and Bothwell^55^, with slight modifications. Briefly, 0.1 g tissue was finely cut and incubated for 20 h in 2 ml of acid solution (3 M HCL, 0.61 M trichloroacetic acid) at 65 °C. After cooling to room temperature, 20 µl of the acid extract was incubated with 200 µl of chromogen solution (0.009% bathophenanthroline sulfonate, 0.09% thioglycolic acid, 45.5% saturated sodium acetate) in a 96 well plate. The OD was measured at 535 nm and concentrations determined with a four-point standard curve ranging from 0 to 0.5 µg of iron and data are reported as total iron in each tissue. For iron staining, spleens were fixed in neutral buffered 10% formalin overnight, dehydrated in ethanol, embedded in paraffin, cut into 5 µm sections, mounted, and iron was detected using the Gomori Prussian Blue Stain Kit (Newcomer Supply, Middleton, WI). Whole-slide images representing entire digitized histopathological tissue sections were scanned using the Panoramic DESK Scanner (3DHistech, Budapest, Hungary) equipped with a 40x (NA 0.95) Carl Zeiss Plan-Apochromat objective lens. Images were captured using CaseViewer (3DHistech, Budapest, Hungary).

### Statistics

Statistical analysis was performed using GraphPad Prism 9. The Student's *t*-test was used for two group comparisons. ANOVA and Bonferroni posttest comparing the selected groups specified in the figure legends was used for studies involving more than two groups. Significance was determined at *p* < 0.05.

## Supplementary Information

Supplementary Information.
