# Supplementary material for: M-CSF supports medullary erythropoiesis and erythroid iron demand following burn injury through its activity on homeostatic iron recycling
Source: Sci Rep. 2022 Jan 24;12:1235. doi: 10.1038/s41598-022-05360-2 (PMC8786861; doi:10.1038/s41598-022-05360-2)
Supplement: Supplementary file 1 — Supplementary Information. [file 41598_2022_5360_MOESM1_ESM.pdf]

M-CSF supports medullary erythropoiesis and erythroid iron demand following burn injury through its activity on homeostatic iron recycling.

John G. Noel[1], Seth W. Ramser[1], Lori Pitstick[1], John P. Bonamer[2], Bryan Mackenzie[2], Katie G. Seu[3], Theodosia A. Kalfa[3], Jose A. Cancelas[4|], Jason C. Gardner[1,*]

[1] University of Cincinnati College of Medicine, Division of Pulmonary, Critical Care and Sleep Medicine, Cincinnati, 45267, USA

[2] University of Cincinnati College of Medicine, Department of Pharmacology and Systems Physiology, Cincinnati, 45267, USA

[3] Cincinnati Children's Hospital Medical Center, Cancer and Blood Diseases Institute, Cincinnati, 45229, USA

[4] Cincinnati Children's Hospital Medical Center, Divisions of Pathology and Experimental Hematology and Cancer Biology, Cincinnati, 45229, USA

* Corresponding. gardnejr@uc.edu

Supplementary Table 1. RT-PCR Primers

Gene	Forward	Reverse
Fpn1	ATGGGAACTGTGGCCTTCAC	TCCAGGCATGAATACGGAGA
Spic	CGTACAGAACATAGCTGAAAGC	TGTCAACCCACTGAATACAAGA
Hmox1	TTACCTTCCCGAACATCGAC	CTAGCAGGCCTCTGACGAAG
Hamp	TTGCGATACCAATGCAGAAGA	GATGTGGCTCTAGGCTATGTT
Actb	GACTCATCGTACTCCTGCTTG	GATTACTGCTCTGGCTCCTAG
Hprt	CCCCAAAATGGTTAAGGTTGC	AACAAAGTCTGGCCTGTATCC

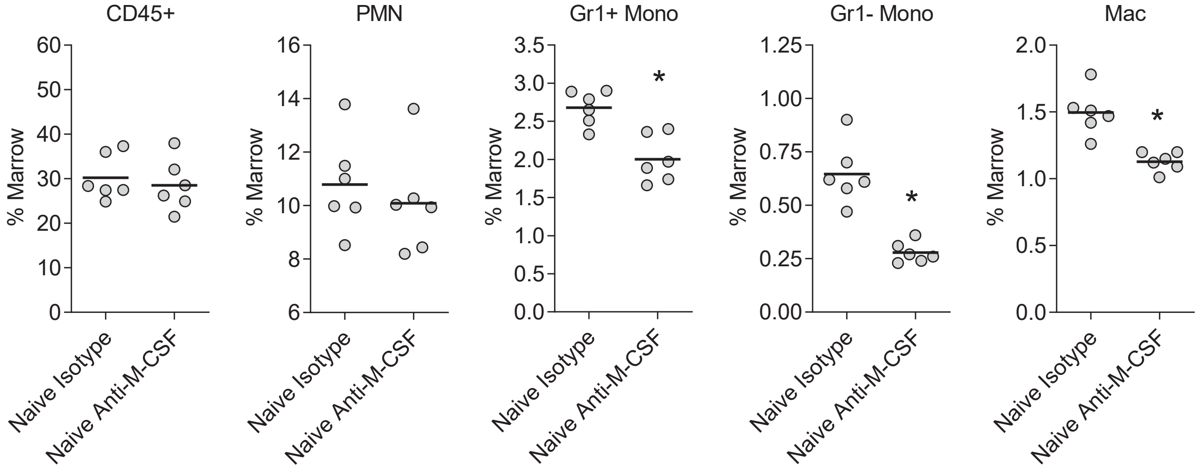

Supplementary Figure 1. Effect of M-CSF neutralization on myeloid cells in the bone marrow of naive mice. Non-injured mice were administered isotype or M-CSF neutralizing antibodies on day 0, then daily for six days, and bone marrow cells were collected day seven for analysis using flow cytometry as in other studies. A t-test was used to compare isotype vs anti-M-CSF groups, *p<0.05.

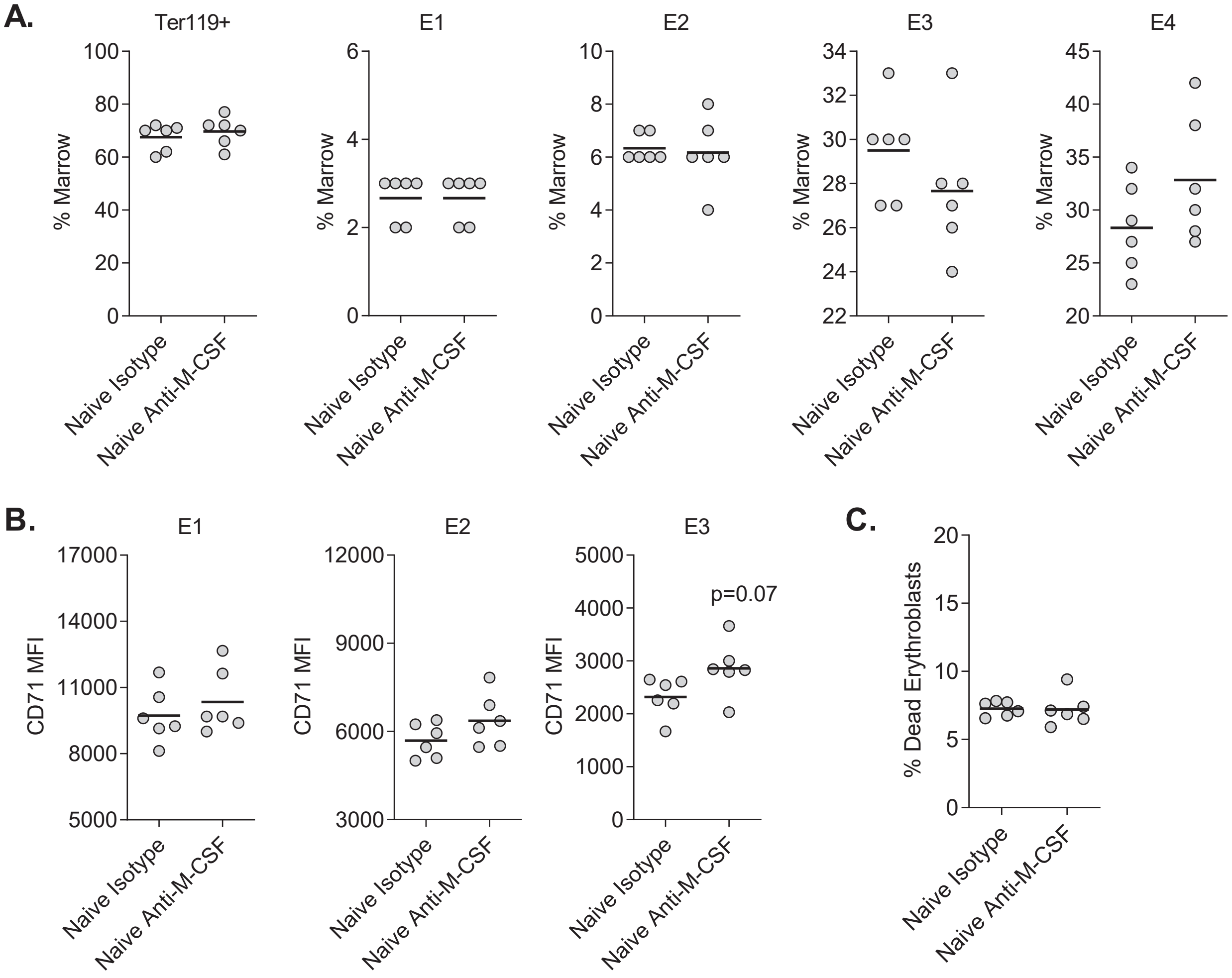

Supplementary Figure 2. Effect of M-CSF neutralization on erythroid cells in the bone marrow of naive mice. Non-injured mice were administered isotype or M-CSF neutralizing antibodies on day 0, then daily for six days, and bone marrow cells were collected day seven for analysis using flow cytometry as in other studies. (A) Erythroid populations. (B) CD71 MFI of erythroid populations E1 to E3. (C) Percentage of dead erythroblasts. A t-test was used to compare isotype vs anti-M-CSF groups, *p<0.05.

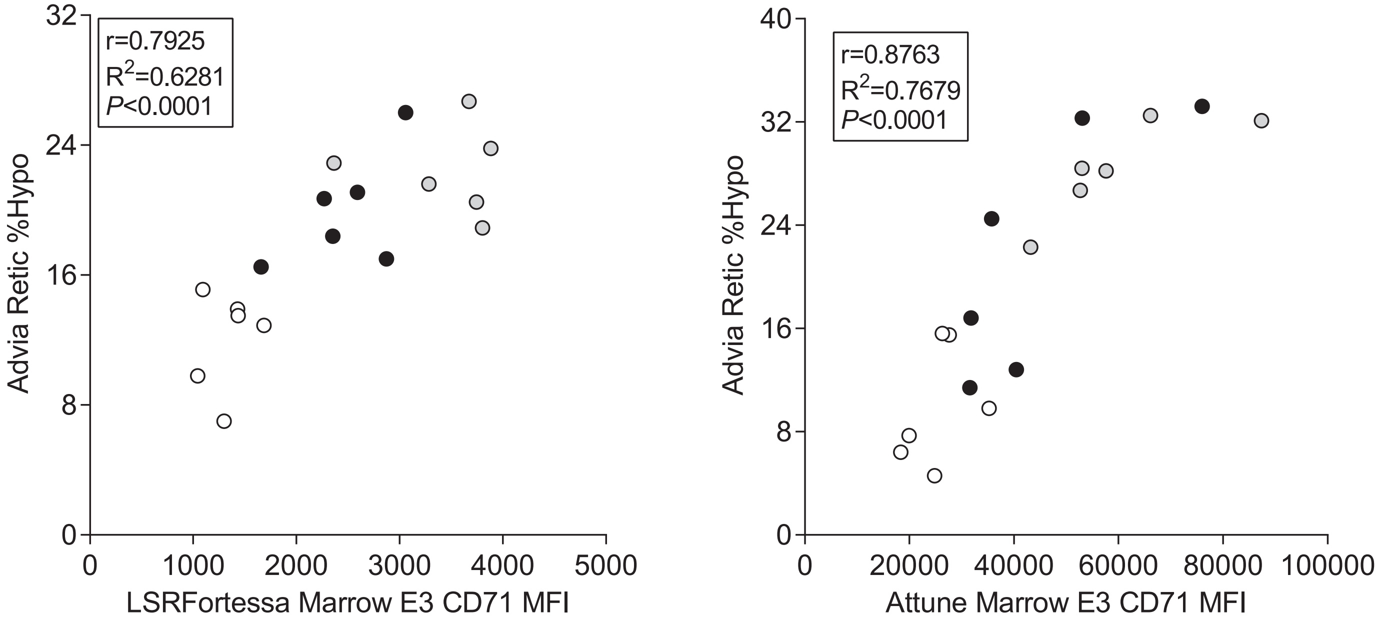

Supplementary Figure 3. Correlation of hypochromic reticulocytes in circulation and transferrin receptor levels on bone marrow erythroid cells. Blood and bone marrow cells were harvested on post injury day seven in two independent M-CSF neutralization experiments. The proportion of hypochromic reticulocytes in circulation was determined on an Advia automated hematology system and CD71 staining intensity on bone marrow erythroid population E3 was determined on an LSRFortessa or Attune flow cytometer. (Left Panel) Correlation of hypochromic reticulocytes vs E3 CD71 MFI determined on an LSRFortessa. (Right Panel) Correlation of hypochromic reticulocytes vs E3 CD71 MFI determined on an Attune. Data points for each experiment are colored according to group; white for sham isotype, black for burn isotype, and light gray for burn anti-M-CSF. Pearson correlation was used for analysis and statistics are shown in the inset.

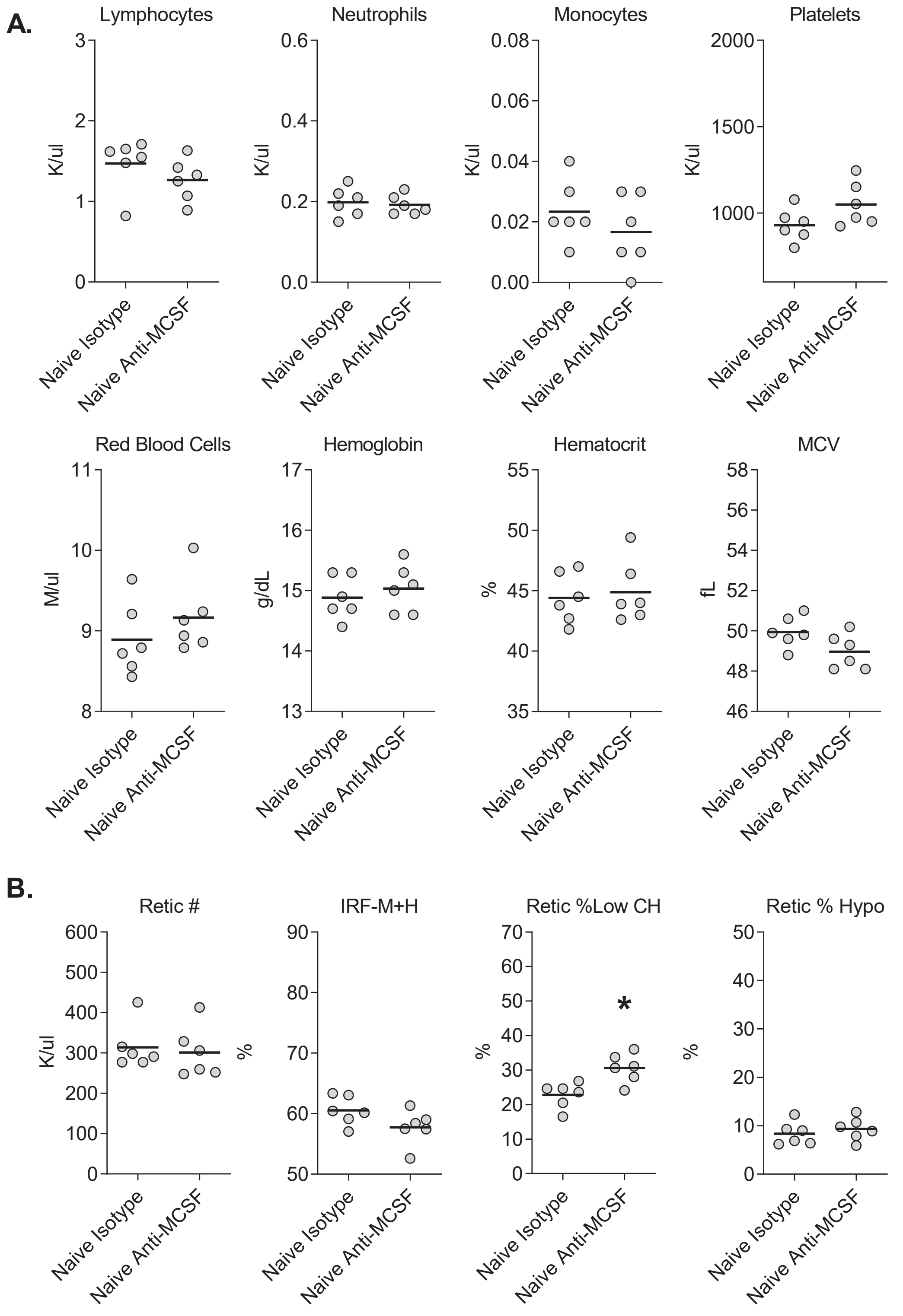

Supplementary Figure 4. Effect of M-CSF blockade on circulating cells in naive mice. Non-injured mice were administered isotype or M-CSF neutralizing antibodies on day 0, then daily for six days, and blood was collected on day seven for analysis on an automated hematology system as in other studies. (A) Blood cell data. (B) Reticulocyte data. A t-test was used to compare isotype vs anti-M-CSF groups, *p<0.05.

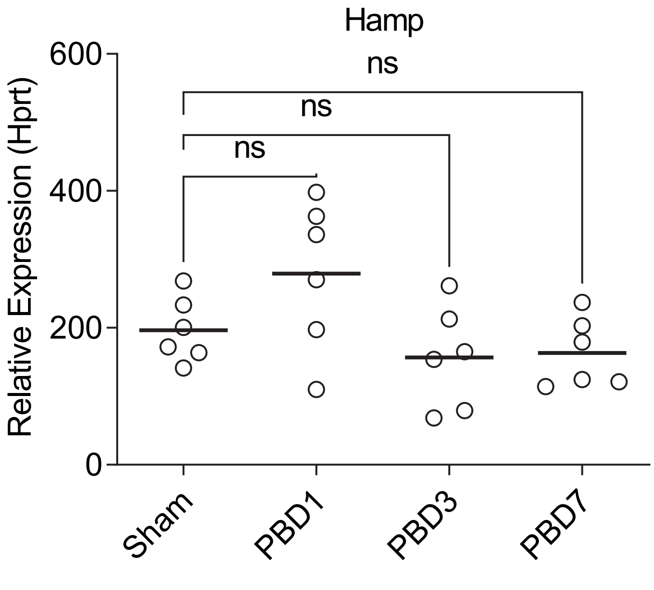

Supplementary Figure 5. Liver hepcidin expression over one week post burn. Livers were harvested from sham injured mice and burn injured mice on post burn day (PBD) 1, 3, and 7 for RT-PCR to quantify hepcidin expression relative to the houskeeping gene Hprt. Not significant (ns) all post burn days compared to sham, ANOVA.
